# Methotrexate plus or minus cetuximab as first‐line treatment in a recurrent or metastatic (R/M) squamous cell carcinoma population of the head and neck (SCCHN), unfit for cisplatin combination treatment, a phase Ib‐randomized phase II study Commence

**DOI:** 10.1002/hed.26053

**Published:** 2020-01-06

**Authors:** Janneke C. Ham, Esther van Meerten, W. Edward Fiets, Laurens V. Beerepoot, Frank J. F. Jeurissen, Marije Slingerland, Marianne A. Jonker, Olga Husson, Winette T. A. van der Graaf, Carla M. L. van Herpen

**Affiliations:** ^1^ Department of Medical Oncology Radboud University Medical Center Nijmegen HB The Netherlands; ^2^ Department of Medical Oncology Erasmus MC Cancer Institute Rotterdam GD The Netherlands; ^3^ Department of Internal Medicine Medical Center Leeuwarden Leeuwarden AD The Netherlands; ^4^ Department of Internal Medicine Elisabeth TweeSteden Hospital Tilburg AD The Netherlands; ^5^ Department of Internal Medicine Medical Center Haaglanden The Hague VA The Netherlands; ^6^ Department of Medical Oncology Leiden University Medical Center Leiden ZA The Netherlands; ^7^ Biostatistics, Radboud Institute for Health Sciences Radboud University Medical Center Nijmegen HB The Netherlands; ^8^ Department of Psychosocial Research and Epidemiology Netherlands Cancer Institute Amsterdam CX The Netherlands; ^9^ Division of Clinical Studies Institute of Cancer Research London UK; ^10^ Department of Medical Oncology Netherlands Cancer Institute Amsterdam CX The Netherlands

**Keywords:** cetuximab, first‐line, methotrexate, palliative treatment, recurrent or metastatic squamous cell carcinoma of the head and neck

## Abstract

**Background:**

Methotrexate in recurrent or metastatic (R/M) squamous cell carcinoma of the head and neck (SCCHN) has limited progression‐free survival (PFS) benefit. We hypothesized that adding cetuximab to methotrexate improves PFS.

**Methods:**

In the phase‐Ib‐study, patients with R/M SCCHN received methotrexate and cetuximab as first‐line treatment. The primary objective was feasibility. In the phase‐II‐study patients were randomized to this combination or methotrexate alone (2:1). The primary endpoint was PFS. Secondary endpoints were overall survival (OS), toxicity, and quality of life (QoL).

**Results:**

In six patients in the phase‐Ib‐study, no dose limiting toxicities were observed. In the phase II study, 30 patients received the combination and 15 patients methotrexate. In the phase‐II‐study median PFS was 4.5 months in the combination group vs 2.0 months in the methotrexate group (HR 0.37; *P* = .002). OS, toxicity, and QoL were not significantly different.

**Conclusion:**

Cetuximab with methotrexate improved PFS without increased toxicity in R/M SCCHN‐patients.

## 1 INTRODUCTION

Head and neck cancer is the seventh most common cancer type worldwide.^1^ Cure rates of patients with locoregionally advanced squamous cell carcinoma of the head and neck (SCCHN) vary between 30% and 60%, and in case of metastases or local recurrence, palliative treatment is often the only option.^2^ In this setting, the only approved treatment with significant overall survival (OS) benefit at the time this study was initiated was cetuximab added to platinum/5FU (OS of 10.1 months vs 7.4 months with platinum/5FU alone), but its toxicity is considerable.^3^ Other treatment options were single agent methotrexate (MTX) or docetaxel in patients unfit for platinum, or combination therapies of platinum combined with 5FU or taxane.^4^ MTX has a response rate (RR) of 10% to 25%, and a mean OS of 6 to 8 months,^5^, ^6^, ^7^, ^8^, ^9^, ^10^ while combination therapy with platinum and 5FU or taxane has RRs of 45% to 50%, but without OS benefit compared with single agent treatment, and with more toxicity.^4^, ^8^, ^10^, ^11^

The epidermal growth factor receptor (EGFR) has shown to be upregulated in 90% to 100% of the head and neck cancers.^12^ Cetuximab is a recombinant, human/mouse chimeric monoclonal antibody that binds specifically to EGFR and inhibits receptor activation by competing with epidermal growth factor. It is approved in the United States as single agent after failure to platinum‐based therapy, based on a single‐arm phase II study with a RR of 13% and median OS of 6 months.^13^

No data are available on the combination of MTX and cetuximab. This combination could be beneficial for patients unfit for or unwilling to get platinum‐based therapy in the recurrent or metastatic (R/M) setting. The aim of this study was to investigate in first‐line R/M SCCHN patients the feasibility of adding cetuximab to MTX and to investigate whether the combination can improve progression‐free survival (PFS) vs MTX alone. Secondary aims were to investigate the OS, RR, toxicity, and health‐related quality of life (HRQoL).

## 2 METHODS

### 2.1 Patients

Patients aged ≥18 years with previously untreated R/M SCCHN, who were unfit for or unwilling to get platinum‐based chemotherapy, were eligible. Other inclusion criteria were at least one measurable lesion as determined by RECIST v1.1., time between prior treatment for locally advanced disease and inclusion in the study of at least 3 months, WHO performance status 0 to 2, and adequate organ function and laboratory tests. Main exclusion criterion was prior treatment with EGFR‐inhibitors or MTX.

The study was approved by the Medical Ethical Research Committee of Radboudumc, the Netherlands and in accordance with the Declaration of Helsinki. All patients signed written informed consent. The http://clinicaltrials.gov Identifier is NCT02054442.

### 2.2 Study design of the Commence study

First, a phase Ib open‐label non‐dose‐escalating study was performed to determine the safety and tolerability of the combination of cetuximab and MTX. Six patients should be treated first and if 0 to 1 dose limiting toxicities (DLT) in these six patients would occur, a phase II study could start.

In the phase II study, patients were randomized between cetuximab and MTX or MTX alone. Participating institute, performance status (0 or 1 vs 2) and local or locoregional recurrence independently of distant metastatic disease (yes vs no) were used as stratification factors.

During treatment patients were seen every week for the first 4 weeks and thereafter every 2 weeks. At these visits adverse events (AEs) (scored by Common Terminology Criteria for Adverse Events [CTCAE] version 4.0), and laboratory measures were recorded. Patients were monitored for AEs during, and for 30 days after the last administration of study medication.

Tumor assessment, that is, CT or MRI of the head and neck, and CT‐scan of the thorax, was performed at baseline and every 8 weeks. HRQoL was assessed at baseline, after 8 weeks, after 24 weeks, after 1 year and at progressive disease using the 30‐item core European Organization for the Research and Treatment of Cancer Quality‐of‐Life Questionnaire (EORTC QLQ‐C30),^14^ the EORTC QLQ Head and Neck Cancer‐Specific Module (EORTC H&N35),^14^ and the Performance Status Scale for Head & Neck cancer patients (PSS‐HN).^15^

In oropharyngeal cancer patients human papillomavirus (HPV) positivity was determined with immunohistochemical staining p16.^16^

### 2.3 Treatment

In the phase Ib study, all patients were treated with cetuximab and MTX. The dosage of MTX was 40 mg/m^2^ weekly, delivered in 5 to 10 minutes iv. The dosage of cetuximab was 400 mg/m^2^ in a 2‐hour infusion for the first infusion, followed by 250 mg/m^2^ in 1 hour, weekly.

Treatment was continued until progressive disease (PD), unacceptable toxicity or refusal by the patient.

In case, despite standard precautions, grade 1 or 2 cetuximab‐related hypersensitivity reaction occurred, infusion rate was reduced or stopped temporarily. In case of cetuximab‐related grade 3 or 4 toxicity, cetuximab had to be discontinued permanently, but continuation of MTX was allowed. In case of MTX‐related grade 4 toxicity in patients treated with the combination, cetuximab could be continued. If the absolute neutrophil count was <1.5 × 10^9^/L and/or thrombocytes <100 × 10^9^/L, MTX had to be postponed for 1 week and if recovered, folinic acid needed to be prescribed.

### 2.4 Endpoint

The primary endpoints in phase Ib were toxicity and the incidence of DLTs after start of treatment. The primary endpoint of the phase II part of the study was PFS, defined as the time from randomization to PD or death. Secondary endpoints for the phase Ib and phase II study were OS (time from randomization to death), RR according to RECIST v1.1 (ie, complete or partial response), the clinical benefit rate (ie, complete or partial response or stable disease), toxicity according to CTCAE v4.0, HRQoL, and HPV‐status in relation to these outcomes.

### 2.5 Statistical analysis

In the phase Ib study, a minimum of six patients were needed, depending on the occurrence of DLTs. The results are summarized using simple descriptive statistical methods.

In the phase II study, 57 patients were needed in each treatment group to achieve 80% power at a .05 significance level to detect a hazard ratio of 0.6 assuming that the addition of cetuximab to MTX would improve PFS by 2 months from 3 months in the MTX alone group to 5 months in the combination group.

Unfortunately, due to financial constraints in the Netherlands in July 2015, after inclusion of six patients in the phase Ib study and 12 patients in the phase II study, the study had to be amended to a design with a total of 45 patients with a 2:1 randomization in the phase II study (30 patients in the MTX with cetuximab group and 15 patients in the MTX alone group). Kaplan‐Meier curves were plotted and PFS and OS were compared by the log‐rank test. Cox‐regression models were fitted to estimate and test hazard ratios. Nonparametric tests were performed using the chi‐square test to test for differences in patient characteristics and occurrence of AEs between the treatment groups. All statistical tests were two‐sided, with an alpha level of 5% considered as statistically significant. HRQoL was only investigated and described exploratively.

## 3 RESULTS

### 3.1 Patient characteristics

**Figure 1:**
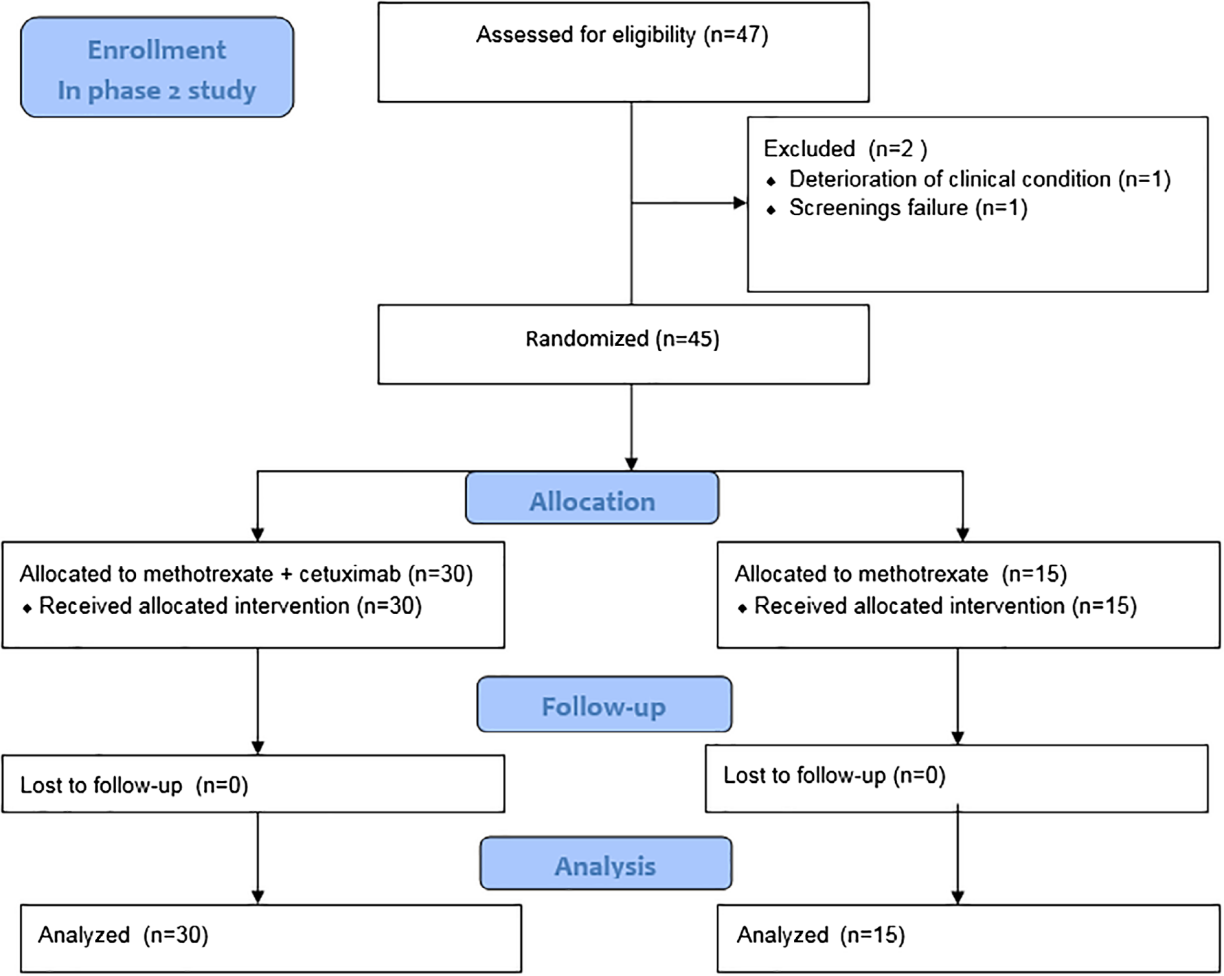
CONSORT flow diagram of the enrollment of patients in the phase II study [Color figure can be viewed at http://wileyonlinelibrary.com]

From February to June 2014, six patients were included in the phase Ib study in the Radboudumc, the Netherlands. All patients received the combination of cetuximab with MTX. The phase II study started in July 2014, but was on hold from July 2015 until August 2016 due to amendment of the study design. Last patient was included in January 2018. Forty‐five patients were included in the phase II study in six participating hospitals in the Netherlands, of which 30 patients were allocated to cetuximab and MTX and 15 patients to MTX alone. (Figure 1). The baseline characteristics were well balanced between the two treatment groups. (Table 1).

**Table 1:** Patient characteristics

	Phase 1 (no. of patients = 6)	Phase 2 MTX + cetuximab (no. of patients = 30)	Phase 2 MTX (no. of patients = 15)	*P* value^a^
Age (median, range)	65.0 (58‐71)	68.5 (46‐80)	64.0 (49‐77)	
Sex				.81
Male (%)	5 (83.3)	23 (76.7)	11 (73.3)	
Female (%)	1 (16.7)	7 (23.3)	4 (26.7)	
WHO				.61
0	4 (66.7)	6 (20.0)	5 (33.3)	
1	2 (33.3)	21 (70.0)	9 (60.0)	
2	0	3 (10.0)	1 (6.7)	
Tumor site				.21
Oral cavity	1 (16.7)	11 (36.7)	2 (13.3)	
Oropharynx	2 (33.3)	8 (26.7)	7 (46.7)	
*HPV positive*	*0*	*4*	*1*	
*HPV negative*	*2*	*3*	*5*	
*HPV unknown*	*0*	*1*	*1*	
Hypopharynx	1 (16.7)	7 (23.3)	2 (13.3)	
Larynx	2 (33.3)	4 (13.3)	4 (26.7)	
Loco‐regional recurrence^b^				.43
Yes	6 (100)	25 (83.3)	11 (73.3)	
No	0 (0)	5 (16.7)	4 (26.7)	
Distant metastases				
Yes	5 (83.3)	21 (70)	7 (46.7)	.13
No	1 (16.7)	9 (30)	8 (53.3)	

a The *P*‐values are for the differences between the treatment groups in the phase II study.

b Loco‐regional recurrence means local recurrence and/or metastases in locoregional lymphnodes.

### 3.2 Efficacy

**Figure 2:**
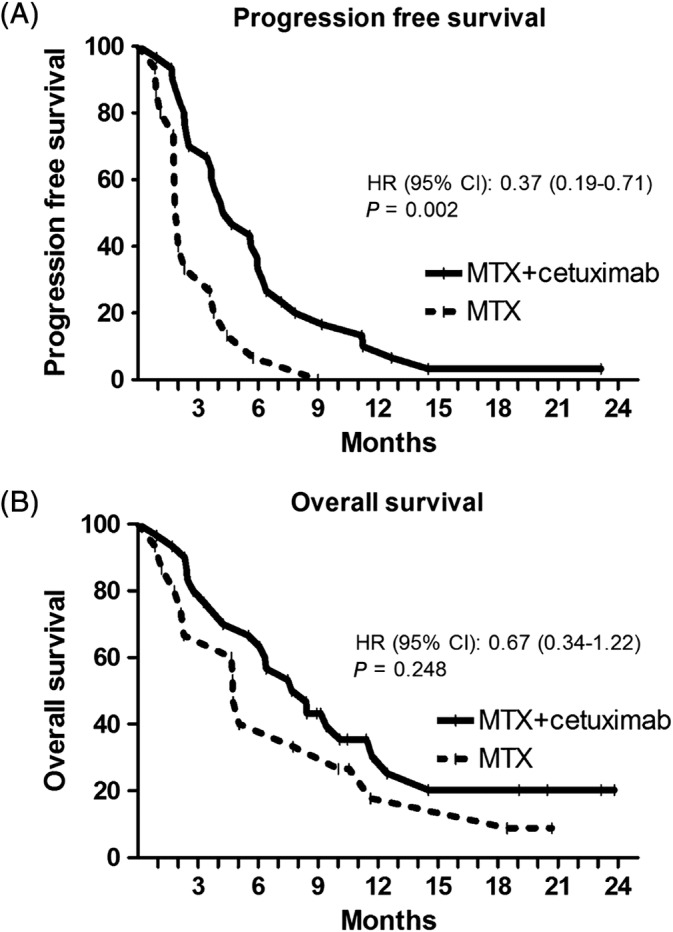
Kaplan–Meier estimates of progression free survival (A) and overall survival (B) according to the two treatment groups

In the phase Ib study, median PFS was 5.8 months (range 1.9‐13.0 months) and median OS 10.6 months (range 3.0‐17.9 months). The cutoff date for the efficacy analysis of the phase II study was October 1, 2018. Median follow‐up was 19.3 months, with a minimum follow‐up of 8.9 months. Median PFS was 4.5 months (range 0.9‐23.2+ months) in the cetuximab and MTX group and 2.0 months (range 0.9‐9.0 months) in the MTX alone group (hazard ratio for progression, 0.37; 95% CI, 0.19 to 0.71; *P* = .002) (Figure 2A). One patient in the combination group, who had a resection of one lymph node metastasis that was growing during treatment without any progression of other lesions, was still on treatment with no signs of progressive disease. The main reason for discontinuation of treatment was PD in both groups.

The median OS in the phase II study was 8.0 months (range 0.9‐23.8+ months) in the cetuximab and MTX group compared with 4.7 months (range 0.9‐20.7+) in the MTX alone group (hazard ratio for death 0.67; 95% CI, 0.34 to 1.22; *P* = .25) (Figure 2B). Eight and two patients were still alive in the combination group and MTX group, respectively. The addition of cetuximab to MTX improved the clinical benefit rate significantly from 40.0% to 76.7% (*P* = .02). The RR showed no significant differences with 13.3% PR in the MTX and cetuximab group and 6.7% PR in the MTX alone group.

### 3.3 Toxicity

In the phase Ib study no DLT's occurred and one serious adverse event (SAE) was reported, not related to the study medication. Three patients reported grade 3 toxicity (two patients had a hypophosphatemia and one syncope), no grade 4 toxicity was observed.

In the phase II study, the overall incidence of grade 3 or 4 AEs was 46.7% in the MTX and cetuximab group compared with 53.3% in the MTX group (*P* = .67). Only the incidence of all grade skin AEs was significantly higher in the combination group compared with the MTX group (86.7% vs 40.0%, *P* = .001). The incidence of dysphagia (16.7% in the combination group vs 46.7% in the MTX group, *P* = .03) and dyspnea (10.0% in the combination group vs 46.7% in the MTX group, *P* = .01) were significantly higher in the MTX group (Table 2).

**Table 2:** Most relevant and common (related and not related) adverse events according to the CTCAE 4.0

	MTX + cetuximab (no. of patients = 30)		MTX (no. of patients = 15)		
	Any grade	Grades 3‐4	Any grade	Grades 3‐4	*P* value^a^
Any event	30 (100)	14 (46.7)	15 (100)	8 (53.3)	.67
Skin reactions^b^	26 (86.7)	3 (10.0)	6 (40.0)	0 (0)	**.001**
Mucositis	14 (46.7)	1 (3.3)	5 (33.3)	1 (6.7)	.39
Xerostomia	3 (10.0)	0 (0)	0 (0)	0 (0)	.21
Dysphagia	5 (16.7)	2 (6.7)	7 (46.7)	2 (13.3)	**.03**
Dyspnea	3 (10.0)	1 (3.3)	7 (46.7)	2 (13.3)	**.01**
Cough	9 (30.0)	0 (0)	5 (33.3)	0 (0)	.82
Pneumonia	5 (16.7)	2 (6.7)	2 (13.3)	0 (0)	.77
Pneumonitits	1 (3.3)	1 (3.3)	0 (0)	0 (0)	.48
Infusion reaction	6 (20.0)	0 (0)	0 (0)	0 (0)	.06
Vomiting	4 (13.3)	0 (0)	1 (6.7)	0 (0)	.50
Diarrhea	10 (33.3)	0 (0)	1 (6.7)	0 (0)	.05
Anorexia	7 (23.3)	0 (0)	4 (26.7)	2 (13.3)	.81
Weight loss	3 (10)	1 (3.3)	0 (0)	0 (0)	.21
Anemia	3 (10)	0 (0)	0 (0)	0 (0)	.21
Thrombocytopenia	3 (10)	1 (3.3)	2 (13.3)	0 (0)	.74
Leucocytopenia	2 (6.7)	0 (0)	0 (0)	0 (0)	.31
Neutropenia	3 (10.0)	0 (0)	0 (0)	0 (0)	.21
Hypercalcemia	1 (3.3)	0 (0)	3 (20.0)	1 (6.7)	.06
Hypomagnesemia	4 (13.3)	0 (0)	0 (0)	0 (0)	.14
Hypophosphatemia	1 (3.3)	1 (3.3)	0 (0)	0 (0)	.48
Hyponatremia	2 (6.7)	2 (6.7)	1 (6.7)	0 (0)	>.99
Hypokalemia	1 (3.3)	0 (0)	0 (0)	0 (0)	.48
Hepatotoxicity	6 (20.0)	4 (13.3)	1 (6.7)	1 (6.7)	.24
Pain tumor	6 (20.0)	0 (0)	2 (13.3)	0 (0)	.58
Pain nontumor	12 (40.0)	0 (0)	5 (33.3)	0 (0)	.66
Tumor hemorrhage	4 (13.3)	0 (0)	0 (0)	0 (0)	.14
Fatigue	16 (53.3)	2 (6.7)	9 (60.0)	2 (13.3)	.67
Depression	3 (10.0)	0 (0)	2 (13.3)	0 (0)	.74
Malaise	9 (30.0)	0 (0)	4 (26.7)	0 (0)	.82
Cardiac event	2 (6.7)	0 (0)	0 (0)	0 (0)	.31

a The *P* values are for the differences between the treatment groups for any grade toxicity, except for the difference in any event, where the *P* value is for the difference between grade 3–4 toxicity.

b Skin reactions included fissures, rash acneiform, rash macula‐papular, paronychia, blisters, nail changes, xerodermia, lymph edema, and toxicity of the eyes.

*P* value of <0.05 is consider as statistically significant.

The total number of SAEs was 14 (in 12 out of 30 patients) in the combination group, compared to eight (5 out of 15 patients) in the MTX group. None of the SAEs in the MTX group was related to MTX, while 3 of the 14 in the combination group were considered as possibly related to MTX or cetuximab. These three (possibly) related SAEs were pneumonia (grade 3), pneumonitis (grade 3), and an infusion‐related reaction (grade 1).

### 3.4 HRQoL

At baseline, 93.3% and 86.7% of the patients completed the HRQoL questionnaires in the cetuximab and MTX group and MTX group, respectively. Only small clinical important differences were found between the two treatment groups at baseline. The compliance of completing the questionnaires during the study as well as at PD was low (Table 3). The HRQoL did not seem to deteriorate after the start of cetuximab and MTX.

**Table 3:** Scores in HRQoL at baseline, after 8 weeks of treatment, and at progressive disease measured by the EORTC QLQ‐C30, QLQ‐H&N35, PSS‐HN, and Visual Analog Scale (VAS)‐score. (N) after each subscales means the number of available questionnaires

	Baseline			After 8 weeks			At progressive disease		
	MTX ± cetuximab (no. of patients = 28)	MTX (no. of patients = 13)	*∆*	MTX ± cetuximab (no. of patients = 16)	MTX (no. of patients = 3)	*∆*	MTX ± cetuximab (no. of patients = 4)	MTX (no. of patients = 2)	*∆*
**PSS‐HN**									
Normalcy of diet (N)	54.4 (27)	49.2 (12)	5.2	70.8 (13)	53.3 (3)	17.5	30.0 (3)	40.0 (2)	−10.0
Eating in public (N)	63.0 (23)	83.3 (12)	−20.3	81.8 (11)	41.7 (3)	40.1	62.5 (2)	62.5 (2)	0.0
Understandability of speech(N)	78.9 (26)	68.8 (12)	10.1	75.0 (13)	66.7 (3)	8.3	83.3 (3)	50.0 (2)	33.3
VAS‐score (N)	2.3 (26)	3.4 (13)	−1.1	2.3 (26)	3.4 (13)	−1.1	3.2 (5)	3.3 (2)	−0.1
**QLQ‐C30**									
Physical functioning	79.4	86.2	−6.8^a^	75.2	73.3^b^	1.9	45.0	83.3	−38.3
Role functioning	82.7	78.2	4.5	72.9	83.3	−10.4	22.2	58.3	−36.1
Emotional functioning	78.0	79.5	−1.5	80.0	94.4^b^	−14.4	75.0	83.3	−8.3
Cognitive functioning	88.7	92.3	−3.6^a^	82.3	94.4	−12.1	58.3	83.3	−25.0
Social functioning	84.5	89.7	−5.2^a^	79.2	77.8^b^	1.4	50.0	83.3	−33.3
Global health status	67.3	73.1	−5.8^a^	64.6	83.3^b^	−18.7	33.3	70.8	−37.5
Fatigue	23.4	22.2	1.2	28.8	11.1^b^	17.7	52.8	22.2	30.6
Nausea/vomiting	6.5	3.8	2.7	1.0	11.1	−10.1	20.8	0.0	20.8
Pain	22.0	23.1	−1.1	18.8	11.1#	7.7	45.8	42.7	3.1
Dyspnea	13.1	7.7	5.4*	20.8	11.1	9.7	16.7	0.0	16.7
Insomnia	11.9	12.8	−0.9	14.6	11.1	3.5	25.0	50.0	−25.0
Appetite loss	17.3	17.9	−0.6	21.4	0.0^b^	21.4	77.8	0.0	77.8
Constipation	15.5	15.4	0.1	18.8	0.0^b^	18.8	8.3	0.0	8.3
Diarrhea	8.3	10.3	−2.0	8.3	0.0^b^	8.3	16.7	33.3	−16.6
Financial problems	6.0	12.8	−6.8^a^	6.3	0.0^b^	6.3	8.3	0.0	8.3
**QLQ‐H&N35** c									
Pain	23.5	20.1	3.4	17.7	11.1	6.6	45.8	41.7	4.1
Swallowing	24.3	21.2	3.1	21.4	11.1^b^	10.3	27.1	37.5	−10.4
Senses problems	23.2	20.5	2.7	20.8	25.0	−4.2	37.5	50.0	−12.5
Speech problems	24.8	22.2	2.6	28.5	0.0^b^	28.5	41.7	33.3	8.4
Social eating	25.4	16.0	9.4	11.5^b^	33.3^b^	−21.8	63.9	16.7	47.2
Social contact	10.5	4.6	5.9	6.3	2.2	4.1	46.7	33.3	13.4
Sexuality	40.6	35.9	4.7	26.9^b^	11.1^b^	15.8	58.3	50.0	8.3
Teeth	15.3	8.3	7.0	11.1	0.0	11.1	22.2	0.0	22.2
Opening mouth	29.6	35.9	−6.3	25.0	11.1^b^	13.9	41.7	66.7	−25.0
Dry mouth	26.2	33.3	−7.1	20.0	33.3	−13.3	33.3	33.3	0.0
Sticky saliva	35.9	44.4	−8.5	44.4	0.0^b^	44.4	50.0	50.0	0.0
Coughing	29.8	20.5	9.3	35.4	0.0^b^	35.4	25.0	33.3	−8.3
Felt ill	13.1	10.3	2.8	16.7	0.0^b^	16.7	50.0	16.7	33.3
Pain killers	75.0	69.2	5.8	81.3	100.0^b^	−18.7	100.0	100.0	0.0
Nutritional supplements	48.1	30.8	17.3	33.3^b^	66.7^b^	−33.4	25.0	0.0	25.0
Feeding tube	25.0	16.7	8.3	25.0	66.7^b^	−41.7	25.0	50.0	−25.0

Abbreviations: ₣, large clinical important difference; ¥, medium clinical important difference.^17^

a Small clinical important difference.

b Clinical relevant difference of more than 10 points between baseline and 8 weeks after the start of treatment within one group.

c Weight gain and weight loss not reported due to difficult interpretation.

The 16 patients in the combination group who completed the questionnaires at 8 weeks after start of treatment, did not differ much in baseline scores from the patients who did not completed the questionnaires after 8 weeks. The only notable differences were that patients who completed the questionnaires after 8 weeks, felt less ill at baseline and had less problems of a dry mouth.

### 3.5 HPV

Four out of 8 (50%) vs one out of 7 (14.3%) oropharyngeal carcinomas were HPV positive in the combination group and MTX group, respectively. Because of the small number of oropharyngeal cancers in both groups, no conclusions can be drawn about the effect of HPV‐status on PFS and OS.

## 4 DISCUSSION

This phase Ib‐randomized phase II study of first‐line treatment of R/M SCCHN showed a significant increase in PFS by adding cetuximab to MTX with 2.5 months. The clinical benefit rate of 76.7% in the combination group was significantly higher compared with 40.0% in the MTX group.

The 2.5 months benefit in PFS by adding cetuximab to MTX, is in line with earlier studies showing benefits of adding cetuximab to platinum‐based therapy^3^, ^18^; however, this is the first study in combination with MTX. Although Vermorken et al showed a clinically significant difference in OS with adding cetuximab to platinum‐5FU, there was substantial toxicity, with grade 3/4 toxicity rate of 82%, whereas the patients in our study treated with the combination of MTX and cetuximab reported grade 3/4 toxicity in 46.7%. The PFS of 2.0 months in the MTX alone group was slightly less than expected before the start of the study (power calculation was made with the consumption of a PFS of 3.0 months in the MTX alone group). The PFS and OS are in line with other studies comparing MTX with other chemotherapy regimens or tyrosine kinase inhibitors in first and second line (Table 4).

**Table 4:** Overview of studies, in which MTX monotherapy was compared with other chemotherapy regimens or oral tyrosine kinase inhibitors in R/M SSCHN patients

Study	Line of treatment	Regimen	Number of patients	RR	Median PFS (months)	Median OS (months)
Forastiere^8^	First	Cisplatin +5FU	87	32	4.2	6.6
		Carboplatin +5FU	86	21	5.1	5.0
		MTX	88	10	4.1	5.6
Stewart^19^	Any	Gefitinib 250 mg	158	2.7	‐	5.6
		Gefitinib 500 mg	167	7.6	‐	6.0
		MTX	161	3.9	‐	6.7
Kushwaha^6^	Any	Gefitinib 500 mg	39	7.7	‐	8.8
		MTX	40	5.0	‐	7.8
		MTX + 5FU	38	7.9	‐	8.1
Machiels^20^	Second	Afatinib 40 mg	322	10	2.6	6.8
		MTX	161	6	1.7	6.0
Machiels^21^	Second	Cabazitaxel	53	0	1.9	5.0
		MTX	48	0	1.9	3.6
This study	First	MTX + cetuximab	30	13.3	4.5	8.0
		MTX	15	6.7	2.0	4.7

The landscape of the treatment of R/M SCCHN patients is rapidly evolving due to the introduction of immunotherapy. Nivolumab is already registered for patients with R/M SSCHN after platinum‐failure, showing an improved 2‐year OS rate of 16.9% vs 6.0% with investigator's choice chemotherapy (HR 0.68; 95% CI, 0.54‐0.86).^22^ Recently, the results of the KEYNOTE‐048 phase III study were presented in which the current standard cetuximab‐platinum/5FU was compared with pembrolizumab monotherapy or pembrolizumab combined with platinum/5FU in first‐line R/M head and neck cancer. This study showed that pembrolizumab alone improved OS compared with standard chemotherapy (14.9 months vs 10.7 months, HR 0.61, *P* = .001) in patients with ≥20 CPS (combined positive score; PD‐L1 expression in tumor and/or surrounding immune cells, divided by tumor cells).^23^ Despite these developments, there will always be patients who are not suitable for immunotherapy because of auto‐immune diseases or a PDL‐1 negative cancer and who are too vulnerable for treatment with platinum‐based chemotherapy. For these patients, the combination of MTX and cetuximab, as it has shown a PFS benefit at the price of limited and well manageable toxicities, can be an interesting treatment option. New studies in which this combination can be compared in first and/or second‐line therapy in patients unfit for, or after failure of immunotherapy would increase the insight in this combination treatment.

Limitations of this study are the small number of patients included and the altered study design. Therefore, the results and statistical analyses should be interpreted with caution and should be seen as hypothesis generating for new studies in the future.

Another limitation is the small number of completed HRQoL questionnaires, which made it difficult to draw firm conclusions about the effect of adding cetuximab to MTX on HRQoL, while patient reported outcomes are getting more important. However, the fact that the HRQoL did not deteriorate after the start of cetuximab and MTX, is in line with Mesia et al, who showed that adding cetuximab to platinum‐fluorouracil does not adversely affect QoL in patients with R/M SCCHN.^24^ As shown by the rapid decline in HRQoL at PD, a stable HRQoL after start of the treatment is of clinical relevance in this patient group.

In conclusion, in this small study the addition of cetuximab to MTX improved PFS in patients with R/M SCCHN, and could be considered as treatment option in patients not eligible or unfit for platinum‐based therapy.
